# Exploring teacher discourse patterns: Comparative insights from novice and expert teachers in junior high school EFL contexts

**DOI:** 10.1016/j.heliyon.2024.e36435

**Published:** 2024-08-17

**Authors:** Zhiyue Tong, Fengcun An, Yanji Cui

**Affiliations:** School of Foreign Languages, Yanbian University, Yanji, China

**Keywords:** Teacher discourse patterns, Novice teachers, Expert teachers, Junior high schools, EFL contexts

## Abstract

Effective teacher discourse is critical in improving English as a foreign language (EFL) education, particularly in junior high schools in China, where students are at a crucial stage in their language development. As junior high school students are at a pivotal developmental stage, this research investigates the discourse patterns employed by novice and expert teachers to assess their impact on students' engagement and language acquisition. Despite the extensive research on teacher discourse in higher education, a significant gap remains regarding its application in compulsory primary education settings. This study aims to fill this gap by examining the current classroom discourse patterns of EFL teachers in junior high schools to identify the distinctions between novice and expert teachers and explore the factors contributing to these differences. This mixed-methods study includes qualitative and quantitative analyses. Verbatim transcriptions of six classes were used to create a corpus exceeding 20,000 words. The data were analysed using cross-tabulation in Excel and Chi-square tests in SPSS 22.0, complemented by semi-structured interviews with selected teachers. The theoretical framework is grounded in Long's（1996）interaction hypothesis, which underlines the significance of communication in facilitating language proficiency through meaningful interaction, and the analysis follows Sinclair and Coulthard's（1975）discourse patterns. The initiation-response-feedback (IRF) and initiation-response-0 (IR0) emerged as predominant patterns among both novice and expert teachers. Novice teachers predominantly relied on the basic IRF pattern, while expert teachers exhibited greater flexibility and more frequent use of variant patterns, such as IRFR, I[R^n^F^n^] and [I^n^R^n^]F. Such adaptability among expert teachers creates a more interactive and engaging learning environment, thereby enhancing student participation and language acquisition. The study also identifies a novel variant structure, IR^n^F, used more frequently by expert teachers, underlining the benefits of group work in fostering teamwork and independent thinking. Expert teachers demonstrated a greater propensity to adapt their discourse strategies to foster a more production-oriented learning environment, which was the main factor driving the teachers' differing discourse patterns. This study significantly contributes to the analysis of teacher discourse in the junior high school EFL context, providing empirical evidence and practical insights that bridge the gap between theory and practice. By elucidating the distinct discourse practices of novice and expert teachers, this study offers valuable recommendations for teacher professional development and highlights the importance of employing varied and interactive discourse structures to improve EFL teaching effectiveness. The study also provides valuable insights for educators striving to improve their instructional practices and the language acquisition in EFL classrooms.

## 1 Introduction

In recent years, the exploration of teacher discourse in second language acquisition (SLA) has generated significant scholarly interest, particularly within the context of English as a foreign language (EFL) education [1]. The intricate dynamics of classroom interactions and the pivotal role of teacher discourse in facilitating language learning have been extensively studied, illuminating their impact on student engagement, comprehension and overall language proficiency. Despite the wealth of research, there remains a notable gap in our understanding of how teacher discourse varies between novice and expert teachers, especially in junior high school settings in China.

The study of teacher discourse can be traced back to the seminal works of Harris (1952) [2], who introduced discourse analysis and thus laid the foundation for subsequent research in the field, and Sinclair and Coulthard (1975) [3], who developed the initiation-response-feedback (IRF) model, which further refined our understanding of classroom discourse patterns, emphasizing the structured nature of instructor–learner interactions [4]. These foundational studies were instrumental in shaping the discourse analysis framework, providing a lens through which contemporary research continues to evolve. It is essential for non-native speakers to continuously work on maintaining their foreign language proficiency to avoid language attrition [5]. Within the Chinese context, EFL teachers play a vital role in facilitating students’ foreign language learning, especially in basic education, where exam preparation takes precedence [6]. Other related research has explored, for instance, types of questioning in college classrooms [7,8] and the structure of pedagogic discourse [9,10]. However, as these studies have predominantly focused on senior high schools or colleges, the junior high school context—a critical stage of systematic English language learning in China—has been overlooked. Moreover, it is crucial to conduct research on the classroom discourse of junior high school foreign language teachers owing to the significant impact that English can have in fostering sustainable economic growth and national development [11].

Therefore, the authors aimed to fill this gap by conducting a comparative study on the teacher discourse of novice and expert teachers in junior high schools in a city in eastern China. A mixed-methods approach was employed with the primary quantitative analysis involving the use of Excel for cross-tabulation and SPSS 22.0 for Chi-square tests to analyse the teacher discourse patterns. To complement the quantitative findings, qualitative semi-structured interviews were conducted. The qualitative data were analysed thematically to identify common themes and insights explaining the quantitative results. The objective of integrating quantitative and qualitative methods is to produce new insights into IRF and its variant patterns, thereby enhancing our understanding of effective EFL teaching practices.

By addressing the under-explored area of teacher discourse in Chinese junior high schools, this study not only contributes to the existing research but also provides practical implications for teacher professional development. The findings are expected to inform pedagogical strategies and promote a production-oriented approach that fosters learner engagement and language acquisition. Ultimately, this research is intended to support the continuous improvement of EFL education, ensuring that both novice and expert teachers can effectively contribute to learners’ linguistic and cognitive development.

## 2 Working definition of key conceptions over time

### 2.1 Novice and expert teachers

Scholars in China and abroad have different ideas regarding the criteria for defining novice and expert teachers. Examples include years of teaching experience and social recognition.

Berliner [12] outlines five developmental stages through which novice teachers evolve into experts: novice, advanced beginner, competent teacher, proficient teacher and expert teacher. He suggests that novices typically achieve competency after three to four years of experience, with most teachers reaching proficiency by their fifth year and only a few advancing to become experts. Palmer et al. [13] introduce four key criteria to differentiate between proficient and expert teachers: years of experience, social recognition, professional or social group membership and performance-based criteria. Sternberg [14] emphasizes that expert teachers possess extensive expertise in their teaching field and identifies three main characteristics of expert teachers: instructional expertise, high efficiency and creative insight. Expert teachers demonstrate superior abilities in observing classroom behaviour, providing essential language references for learners, employing flexible teaching methods and efficiently processing and interpreting information. They also exhibit a passion for teaching and learning and disseminate knowledge in a creative manner to engage students and enhance their language acquisition, a trait typically lacking in novice teachers.

Numerous researchers have conducted comparative analyses of the teaching efficiency and behaviours of novice versus expert teachers in the Chinese context. Yu [15] asserts that teaching efficiency and behaviour are key factors that differentiate novice from expert teachers. Teaching efficiency involves the teacher's self-assessment of their ability to effectively handle tasks and organize activities. Yu also highlights two distinguishing characteristics of expert teachers, who are expected to demonstrate greater confidence and positivity in their teaching practices compared to novice teachers. Maintaining a positive attitude can enhance students' psychological well-being, while displaying confidence can subtly influence the students' mental and emotional development. Lian [16] categorizes teachers into three groups: novice teachers, proficient teachers and expert teachers. Novice teachers are defined as those with fewer than five years of teaching experience and junior titles, whereas expert teachers have at least 15 years of experience and hold senior titles. Proficient teachers, as described by Berliner [12], fall between novice teachers and experts.

The present study delineates novice and expert teachers according to the specific development criteria outlined by Lian [16] and adjusts for the specific research context as follows. First, regarding years of experience, novice teachers typically have teaching experience ranging from zero to three years and may still be developing their teaching styles and classroom management skills. Conversely, expert teachers possess a minimum of 10 years of experience and demonstrate advanced pedagogical skills and a profound understanding of their subject matter. Second, concerning professional recognition, novice teachers typically hold junior titles or are in the early stages of their teaching careers, whereas expert teachers usually have received senior titles or pedagogic awards, indicating higher levels of professional achievement and recognition in competitive settings. Third, regarding educational background, novice teachers only hold a bachelor's degree or diploma in a related field, whereas expert teachers must possess at least a bachelor's degree or higher.

### 2.2 Teacher discourse

**Fig. 1:**
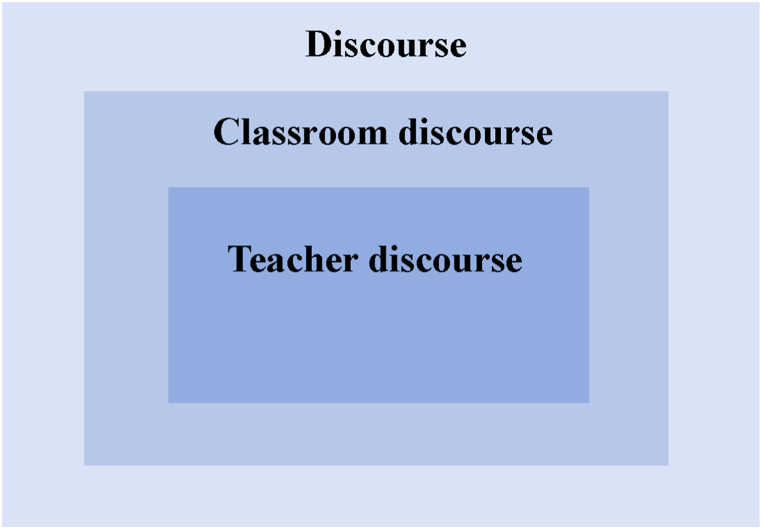
The relationships between discourse types.

Teacher discourse has been a topic of interest among scholars in the SLA field for several decades. The term was first introduced by Harris [2]. VanDijk [17] and Ellis [18] subsequently elaborated on its definition, function and features. Subsequent studies by McCarthy [19], Nunan [1] and Cook [20] have further explored the concept. According to Sinclair and Coulthard [3], teacher discourse refers to the artificial or stylized language used by second or foreign language instructors to convey information to learners. Ellis [18] highlights that teacher discourse directed at second language learners is considered a specific register with unique formal and interactional properties. McCarthy [19] categorizes teacher discourse into classroom discourse, instructional discourse, communicative discourse and feedback discourse. In the Chinese context, Dai and Li [21] propose a rational definition of teacher discourse, positing that teacher discourse provides a straightforward code for delivering comprehensive input to learners in the SLA context. Furthermore, Cheng [22] contends that teacher discourse is a form of language generated by teachers during the organization and execution of teaching. Cheng [22] further elaborates on teacher discourse in the EFL context and depicts the interconnections among these factors as shown in Fig. 1.

### 2.3 Related studies

Scholars abroad have been dedicated to the study of teacher discourse for many decades. Harris [2] pioneered discourse analysis, sparking widespread interest in teacher discourse especially among researchers and linguists. Bellack et al. [23] propose that a classroom should be divided into distinct moves, with each move classified and coded to analyse teaching methods and structures. They introduce a pattern for teacher discourse that emphasizes structuring, soliciting, responding and reacting. Building on this, Sinclair and Coulthard [3] introduce the IRF discourse pattern, highlighting the absence of an information gap between teachers and learners. In this pattern, the teacher initiates to elicit responses from learners and then provides feedback, creating a new conversational turn. Following Sinclair and Coulthard's (1975) [3]framework, Long and Sato [24] propose a classification approach for teacher discourse, categorizing questions into display questions (with fixed answers known to the teachers) and referential questions (open-ended questions with multiple answers that require higher-level thinking from the learners). Chaudron [25] proposes several key elements regarding the teacher's classroom discourse from a linguistic perspective. First, teachers often use a slower speed during EFL classes to facilitate student comprehension. Second, incorporating pauses in the teaching gives the students more time to process the information and excel academically. The more appropriate the use of pauses is, the greater the understanding will be. Third, teachers typically use simple vocabulary and clear pronunciation, rather than complex terminology, when introducing language. Lastly, concerning grammar and discourse, teachers simplify sentence structures and confirm the students' understanding by repeating information. Numerous scholars have conducted extensive empirical research on classroom discourse, including Hakansson [26], Ellis [27], Allwright and Bailey [28], Cullen [29] and Thoms [30].

Numerous studies on teacher discourse in China have been undertaken. Wu [31] analyses turn-taking in EFL classes based on Van Lier's [32] turn-taking transfer system. Zhou and Zhou [8] investigate teacher discourse in English teaching at the college level, focusing on discourse quantity, questioning types, interactional modification and approaches to providing feedback through natural classroom recordings. Their findings reveal that teachers in traditional classrooms tend to adopt an examination-oriented approach, pose display questions and even provide negative feedback, potentially leading to a lack of student engagement, frustration and stifled creativity. Hu et al. [7] investigate the teacher discourse of foreign versus Chinese English teachers, highlighting differences in terms of student talk time, and demonstrate that the foreign teachers' classrooms had significantly higher student talk time—at least double the amount—compared to the Chinese teachers. Li and Fan [33] examine EFL teaching procedures and develop a corpus from classes at various universities to analyse discourse units, ultimately identifying four discourse patterns: IRF (basic pattern), IRFR (teacher modification after student error), IR[I1R1(I2R2)]F (teacher interaction after incorrect responses and before providing feedback) and IR1F1/R2F2 (teacher question followed by student response and then feedback, with potential for continued interaction). Huang [34] introduces a fresh perspective on the advancement of research on IRF classroom discourse patterns. Huang [35] also argues that the IRF framework has emerged as a valuable tool for analysing classroom discourse, leading to a novel interpretation of classroom discourse patterns compared to previous studies.

The existing studies predominantly focus on traditional teacher-centred classroom teaching, where the teachers typically adopt a dominant role and exert significant control over the teaching process. This often results in limited interaction and negotiation between the teacher and the learners. Mahboob [10] argues that a teacher-centred teaching approach can minimize opportunities for learners to express themselves and inhibit their creativity. It is widely acknowledged that skilled teachers should adapt their discourse according to individual learners' abilities to facilitate SLA. Many linguists and scholars have examined teacher discourse from semantic and pragmatic perspectives, emphasizing the importance of the teachers’ language and behaviour. However, enhancing the effectiveness of teaching and learning requires scholars to systematically analyse second language classrooms. While most studies have focused on traditional classroom settings with native English speakers as research participants, there is limited research on teacher discourse in Chinese educational contexts.

Previous studies [7,8,34,35] have tended to focus on senior high schools and colleges rather than junior high schools, although the latter represent a crucial context for Chinese students to systematically learn English. Previous research has predominantly reflected the researchers' viewpoints, resulting in a gap between theory and practical application. Wang et al. [36] propose that many researchers in Chinese have been postgraduates with limited exposure to classroom realities and have primarily focused on theoretical aspects. In contrast, all authors of the present study are teachers, and the first author has eight years of teaching experience in junior high school and involvement in course recordings. The authors thus set out to fill this research gap by conducting interviews with teachers to gain their perspectives. While past researchers have made valuable contributions in terms of quantity and interactive adjustments, there is a need to prioritize discourse quality over quantity. Therefore, an exploration of the discourse structure, such as the IRF and IRFR patterns, with a focus on turn, move and turn-taking, can provide insights into learners’ language learning and cognitive development. Additionally, this comparative study aims to support teacher professional development through an in-depth analysis of teacher discourse structure with a comprehensive understanding of effective discourse strategies within the classroom.

### 2.4 Theoretical basis

Building upon Hatch's (1978) work [37] on the role of conversation in grammar development, Long [38] introduces the interaction hypothesis, which emphasizes the value of face-to-face oral communication in enhancing language proficiency. According to this theory, engaging in conversational interaction facilitates the connection between input, internal learner capacities and output, ultimately aiding learners in comprehending and producing the target language. By engaging in meaningful interactions, learners have more opportunities not only to receive comprehensible input but also to accelerate their output. This framework contributes significantly to the research on SLA. Additionally, collaborative learning environments foster increased motivation and initiative and reduced anxiety among learners. Long [38] stresses the importance of input in language acquisition, highlighting its essential role in the initial stages of language learning. He contends that interaction plays a crucial role in modifying the input and output, making the language input more comprehensible and the instructor's output more accurate. Overall, the interaction hypothesis highlights the significance of both input and output in SLA, advocating for collaborative learning practices to enhance learners' proficiency.

**Fig. 2:**
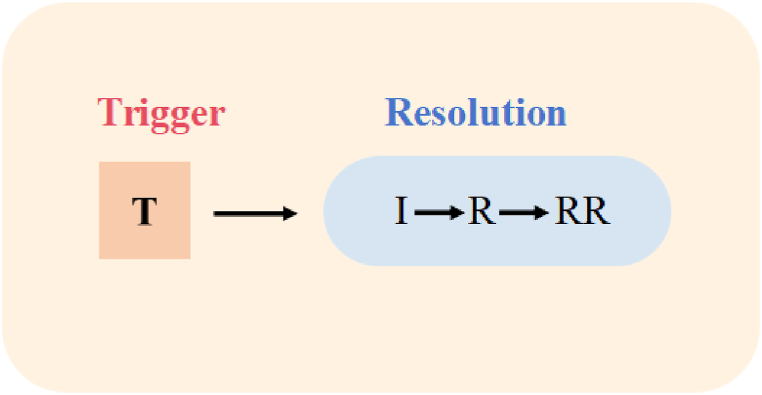
Proposed model for non-understandings.

Long [38] also emphasizes the importance of negotiation of meaning in facilitating language acquisition, particularly when triggered by interactional adjustments made by a native speaker or more competent interlocutor. This negotiation, also known as interactional modification, is aimed at establishing mutual understanding following a breakdown in communication, often due to language comprehension issues between native and non-native speakers. Varonis and Gass [39] outline a structure for the negotiation of meaning, consisting of the trigger, indicator, response and reaction to the response, as illustrated in Fig. 2. The trigger (T) creates the need for negotiation of meaning, proceeding through the stages of indicator (I), response (R) and reaction to the response (RR). This cyclical process continues until mutual understanding is reached, thereby resolving the misunderstanding.

**Fig. 3:**
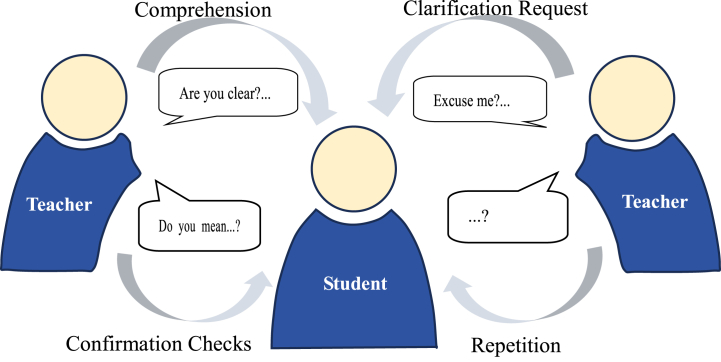
Solutions to communication barriers.

The negotiation of meaning is a critical process in both language acquisition and intercultural communication, where interlocutors collaboratively work towards achieving mutual comprehension. This process is essential for resolving misunderstandings that arise due to linguistic, contextual or cultural differences. The trigger (T) stage involves the initial occurrence of a potential misunderstanding or non-understanding. Triggers may arise from ambiguous language, unfamiliar vocabulary, unclear articulation or complex syntactic structures. The identification of a trigger prompts one interlocutor to seek clarification. At the indicator (I) stage, the listener indicates the presence of a comprehension problem. Indicators can manifest through various communicative strategies, such as requests for repetition, clarification inquiries or expressions of confusion. Then the speaker responds to the listener's indication of the problem by employing several possible strategies or solutions during the response (R) stage: comprehension checks (verifying if the listener has understood the message correctly), clarification requests (providing additional information or context to elucidate the original utterance), confirmation checks (seeking confirmation of specific parts of the message from the listener) and repetition (repeating the original utterance verbatim). Solutions to communication barriers are demonstrated in Fig. 3. At the reaction to the response (RR) stage, the final stage, the listener confirms whether the response has effectively resolved the misunderstanding. This stage can be conveyed through verbal acknowledgments (e.g. “I see”) or non-verbal cues (e.g. nodding, affirmative gestures).

As stated above, the negotiation of meaning entails a dynamic, interactive process whereby interlocutors actively engage in ensuring mutual understanding through the strategies of clarification, repetition, rephrasing and confirmation. This process is indispensable in effective communication, particularly within EFL or SLA contexts.

### 2.5 Descriptive framework

Bellack et al. [23] analyse classroom discourse using Wittgenstein's theory of language games as a theoretical framework. Building on this foundation, Sinclair and Coulthard [3] develop a comprehensive methodology for studying classroom discourse. They propose a descriptive framework for spoken discourse composed of five hierarchical units: act, move, exchange, transaction and lesson. Within this framework, the exchange unit encompasses three distinct moves: initiation, response and feedback, collectively forming a structured sequence known as IRF. This IRF sequence serves as a fundamental characteristic of interactions within classroom discourse:

T: What's the capital of England? (Initiation)

S: London (Response)

T: Yeah, London. Very good. (Feedback)

This IRF module comprises three main components: the teacher initiates a topic (I), followed by student responses (R) concerning the topic and then the teacher's provision of feedback (F) to the students. Despite scholars' differing opinions on the definition of the “F move”, with some viewing it as feedback and others as follow-up, the authors of the present study propose combining feedback and follow-up in the definition of the “F move”. This research examines the IRF exchange pattern in EFL classrooms in the junior high school context from a comparative perspective. Building on the work of Li and Fan [33] and Liu [40], who identify three variation structures within the traditional IRF pattern (i.e. IRFR, [I^n^R^n^]F and I[R^n^F^n^]), this comparative study on novice and expert teachers introduces an additional variation pattern, IR0, through data and corpus analyses.

The IRF (initiation-response-feedback) pattern is widely used to impart knowledge in teacher-oriented classrooms. Within this structure, the teacher poses a question, the student responds and the teacher subsequently evaluates their performance. This process enhances the clarity and flow of communication without altering the meaning or adding supplementary content.

T: How do you pronounce “u-s-h-e-r”? (Initiation)

Ss:/'ʌʃə/(Response)

T: Yeah (Feedback)

The IRFR (initiation-response-feedback-response) pattern is commonly used in teaching settings where a question is posed, followed by a modified answer, feedback and a final response. This pattern involves students imitating or repeating the information provided. Imitation and repetition are recognized as effective learning strategies in SLA as they can improve learning outcomes and enhance engagement in interactive processes.

T: What is this? (Initiation)

Ss: (Unintelligible words) (Response)

T: “Crocodile”. (Feedback)

Ss: “Crocodile”. (Response)

The [I^n^R^n^]F ([initiation-response] … -feedback) pattern involves the teacher posing a question, followed by students responding and then the teacher asking more related questions to keep the discussion going. The teacher provides feedback after several rounds of discussion. This pattern is a simplified variant of the IRF exchange pattern, with no feedback given when the teacher asks a question or assigns a task. Feedback is usually only provided at the end of the final exchange pattern to assess the students’ performance, especially in display questions or role-play scenarios.

T: Have you ever thought of traveling abroad? (Initiation)

S: Yes, I think so. If I have much money … (+++) (Response)

T: Where would you like to go first? (Initiation)

S: I think I like Europe. I think I will go to England and France. (Response)

T: Give us more reasons. (Initiation)

S: These countries have such a long history that they attract me most, and I think, the buildings there are very old and artistic, And I like such things. (Response)

T: Okay, all right. (Feedback)

In the I[R^n^F^n^] (initiation-[response-feedback]- …) pattern, the teacher poses a question or introduces a topic. Then one or many students respond. Next, the teacher offers feedback on the students’ performance without moving on to another topic or question. At the same time, other students may respond to the initial question or topic. Then the teacher gives feedback again. This variation structure can be defined as I[R^n^F^n^], also known as a post-coordinate construction.

T: Extreme sports are to become higher stronger and smarter. Now, if you have to describe a kind of extreme sports, what do you think of right away? (Initiation)

S1: Rock crafting. (Response)

T: A good choice. (Feedback)

S2: Sliding. (Response)

T: Great, yeah! What else? (Feedback)

S3: Surfing. (Response)

T: Yes. I think so too. (Feedback)

The teacher's feedback to the students above is aimed at encouraging continued conversation. This feedback includes simple repetitions, modifications and even extensions in response to the learners' initial answers. The teacher then poses subsequent questions using similar feedback strategies. However, in the following context, the IR0 (initiation-response-0) pattern means that zero feedback is given. This is the practice of the teacher asking a question or initiating a topic and then receiving a response from a learner without providing any further feedback. This type of feedback is commonly seen after a correct response is given, serving as a subtle form of positive reinforcement in the teaching process.

T: What's this? (Initiation)

Ss: Lantern. (Response)

T: (0) (0)

## 3 Method

This study employed a mixed-methods approach incorporating both quantitative and qualitative research methods to comprehensively investigate the discourse patterns of novice and expert teachers in junior high school EFL contexts. The quantitative component of the study involved the statistical analysis of classroom discourse patterns through the use of cross-tabulation in Excel and Chi-square tests in SPSS 22.0. This entailed analysing verbatim transcriptions of six classes, creating a corpus exceeding 20,000 words. The qualitative component was aimed at complementing the quantitative analysis by providing deeper insights into the reasons behind the observed patterns. The semi-structured interviews with selected teachers provided supporting details to explain the quantitative findings. These interviews explored the teachers’ perceptions, experiences and strategies in employing different discourse patterns.

### 3.1 Questions

The research questions guiding this study were as follows:

What are the prevailing discourse patterns employed by EFL teachers in Chinese junior high schools?

What distinctions between the discourse patterns of novice and expert teachers can be identified?

What factors contribute to the disparities in discourse patterns between novice and expert teachers?

### 3.2 Participants

This study utilized purposive sampling [41] and convenience sampling [42] to select as participants three novice and three expert teachers from a city in eastern China. The selection criteria, as outlined in 2.1, included years of experience, professional recognition and educational background. The participants were identified from junior high schools in the eastern province where the researchers study, work and live. The teachers, described in Table 1, have been pseudonymised as ET1, ET2, ET3, NT1, NT2 and NT3 to protect their privacy. To ensure the reliability of this study, the six observed classes were in the listening and speaking section of Unit 3, Section A in Grade 8. Accordingly, the researchers participated in the live recording of all classes and obtained video resources with the instructors’ consent. Each class comprised approximately 50 students, all with comparable levels of English proficiency. These measures were taken to maintain consistency and control variables across the study.

**Table 1:** Relevant participant information.

Participants	Age	Gender	Diploma/Degree	Years of Experience	Professional Recognition
NT1	25	Female	Bachelor	3	Second-grade
NT2	23	Female	Bachelor	1	Second-grade
NT3	28	Female	Master	2	Second-grade
*Average*	*25.3*			*2*	
ET1	41	Female	Bachelor	16	Senior
ET2	43	Female	Bachelor	21	Special-senior
ET3	39	Female	Bachelor	15	Senior
*Average*	*41*			*17.6*	

*Note*：NT, novice teacher; ET, expert teacher.

### 3.3 Instruments

This study adopted the naturalistic inquiry approach [28], which is used primarily to examine the behaviours of teachers and students during classroom engagement. Naturalistic inquiry involves researchers describing and exploring the authentic dynamics of classroom teaching by observing the natural classroom setting. This method allows for conducting an objective analysis as the instructor and learners are observed in their natural environment without any intervention or control, utilizing methods such as classroom observation and semi-structured interviews.

#### 3.3.1 Classroom observation

Richard and Lockhart [43] assert that classroom observation is a highly effective and popular method for understanding the dynamics of a language classroom. The primary goal of classroom observation is to gain insight into the teaching process by observing teaching methods and interactions. The present study entailed recording the teaching procedures of three novice teachers and three expert teachers, with each class session lasting approximately 45 min. The audio recordings were transcribed according to Atkinson's [44] approach as shown in Table 2. Moreover, the classroom interactions were observed without disrupting the natural flow of the teaching process. The corpus analysis and annotation were conducted by the researchers in collaboration with an Associate Professor specializing in SLA at a prestigious university in China. Special attention was given to discourse variants, which were cross-checked and back-annotated for accuracy. The analysis aimed to highlight the distinctive characteristics of novice and expert teachers based on their discourse structures.

**Table 2:** Classroom discourse transcription conventions.

Notation	Meaning
T：	Teacher
L：	Learner（Not identified）
L1, L2, etc.：	Identified learner
LL：	Several learners at once or the whole class
…	Keep silence and look forward to response
(+)	Silence or pause for 1 s
(+)(+)	Silence or pause for 2 s
(+)(+)(+)	Silence or pause for 3 s
(4)	Silence; length given in seconds
?	Rising intonation-question or other
!	Emphatic speech: falling intonation
(hhh)	Laughter
((unintelligible))	Identified speech

**Table 3:** Cross-tabulation of discourse structures of novice versus expert teachers.

			Discourse Structures		Total Numbers
			IRF/IR0	Variants	
Teachers	NTS	Count	80	31	111
		Expected Count	66.5	44.5	111.0
	ETS	Count	86	80	166
		Expected Count	99.5	66.5	166.0
Total Numbers		Count	166	111	277
			
		Expected Count	166.0	111.0	277.0

#### 3.3.2 Semi-structured interview

Two novice and expert teachers were selected to participate in semi-structured interviews respectively based on convenience sampling [42], with each interview lasting at least 20 min. Two teachers were interviewed face-to-face, while one teacher was interviewed via WeChat, an instant messaging platform. The interviews were conducted in Mandarin Chinese by the first author to ensure comprehensive answers. The transcripts were independently coded by the other two authors. Discrepancies were resolved by using an established analytical framework, and the findings were reviewed by the interviewees to ensure clarity and reliability. The interview outline was as follows: (1) In the classroom setting, do students tend to speak more, or does the teacher dominate the conversation? What factors contribute to this dynamic? (2) During lessons, do you frequently utilize a question-and-answer format? If so, what makes this approach effective? Do you employ other interaction strategies in your teaching? (3) What kind of classroom interaction strategy do you consider most beneficial for junior high school students learning a foreign language?

### 3.4 Data collection

Six classes were recorded and transcribed verbatim to create a 20,000-word corpus for research purposes. The transcripts were carefully reviewed and selectively back-annotated to ensure data accuracy. The collected data were then analysed using Excel and SPSS 22.0. First, Excel was used to analyse the data pertaining to the research questions and present the findings through pie graphs, curve charts and mixed charts. Subsequently, SPSS 22.0 was employed to conduct the Chi-square test to explore similarities and differences between the novice and expert teachers in relation to the research questions.

## 4 Results and discussion

**Fig. 4:**
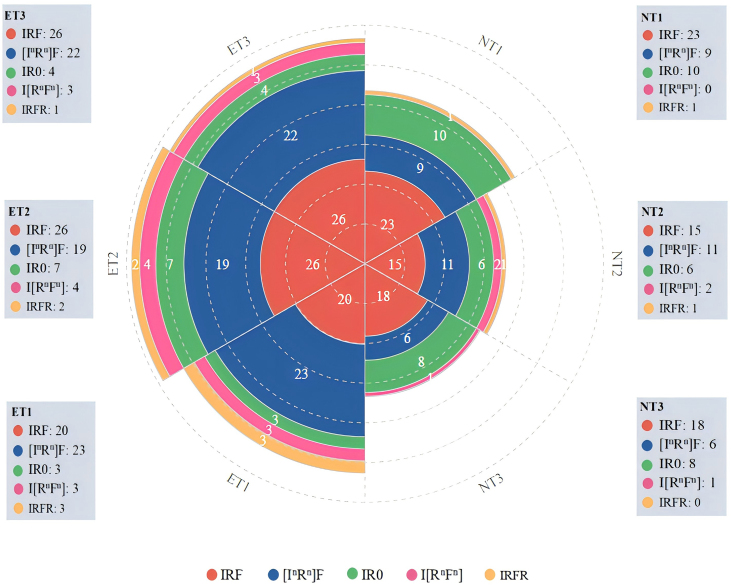
The numbers of different discourse patterns of each teacher.

**Fig. 5:**
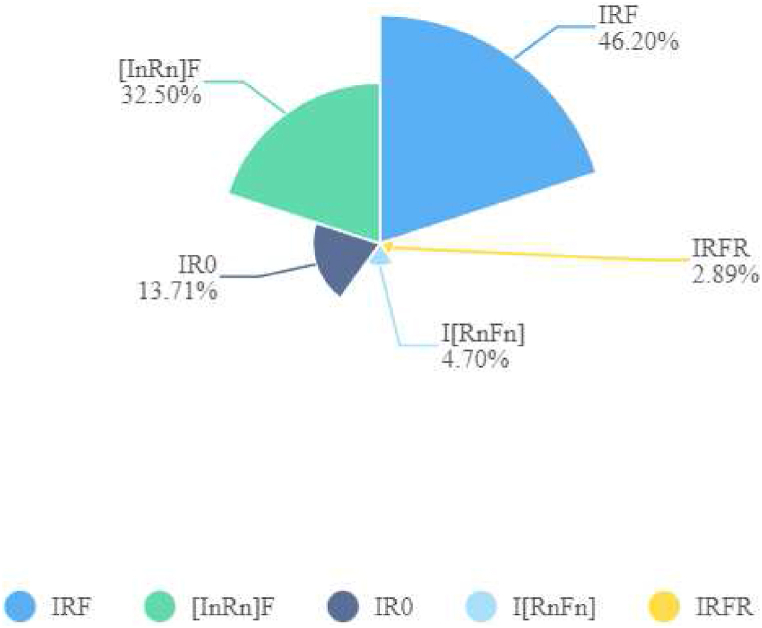
Total numbers of teachers' discourse patterns.

After transcribing the recorded materials, each sentence was meticulously examined to ensure precise statistics. The transcripts were analysed using a detailed coding scheme to capture the various discourse patterns. To maintain the accuracy and reliability of the data, the materials underwent four separate analyses at different time intervals, adhering to established criteria. This thorough process ensured robust findings that could be confidently compared across different teacher groups. Concerning the research questions, Fig. 4, Fig. 5 present the prevailing discourse patterns and distinctions between novice and expert teachers. Specifically, Fig. 4 presents a comparison of discourse patterns and provides detailed data pertaining to both the novice and expert teachers. The comparison highlights significant differences in their use of the IRF pattern and its variants. These differences are crucial in understanding how novice and expert teachers manage classroom interactions and facilitate student engagement.

A comparative analysis of Fig. 4 reveals significant distinctions in the utilization of discourse patterns between novice and expert teachers in junior high school EFL contexts. The IRF pattern dominates across all teachers, with novice teachers NT1, NT2 and NT3 exhibiting 23, 15 and 18 instances, respectively, and expert teachers ET1, ET2 and ET3 demonstrating 20, 26 and 26 instances, respectively. This reveals that the IRF pattern is the prevailing discourse pattern employed by EFL teachers. Fig. 4 also shows that expert teachers demonstrated a more balanced and varied discourse in their interactions compared to novice teachers. This is evidenced by the more prevalent adoption of diverse patterns, such as [I^n^R^n^]F or I[R^n^F^n^], among expert teachers. For the two variant patterns including IRFR, novice teachers NT1, NT2 and NT3 exhibited 10, 14 and 7 instances, respectively, whereas expert teachers ET1, ET2 and ET3 demonstrated 29, 25 and 26 instances, respectively. The frequency of the [I^n^R^n^]F pattern shows that expert teachers tended to engage students in extended discourse more often, promoting deeper interaction and comprehension. They also employed iterative feedback loops more frequently, enhancing student engagement through repeated cycles of response and feedback via the I[R^n^F^n^] pattern. Specifically, these patterns reflect sophisticated levels of interaction and engagement, contributing to a more effective EFL learning environment. The novel pattern, IR0, characterized by initiation and response without subsequent feedback, shows considerable variability among the teachers. Specifically, NT1, NT2 and NT3 demonstrated 10, 6 and 8 instances, respectively, whereas ET1, ET2 and ET3 exhibited 3, 7 and 4 instances, respectively. Overall, the novice teachers exhibited 24 instances of the IR0 pattern, nearly double the total number recorded among expert teachers. This result indicates that the novice teachers might have under-utilized opportunities to provide immediate feedback, indicating potential deficiencies in classroom interaction and student guidance. More distinctions are further elaborated in Fig. 5.

Fig. 5 elucidates the distribution of discourse patterns employed by novice and expert teachers, revealing significant variations during the interactive process. The IRF pattern emerged as the most frequently utilized discourse pattern, making up approximately 46 % of the total. This can be seen in two specific extracts.

[Extract One]

T: What was the girl doing when the rainstorm came? (I)

L: She was doing her homework. (R)

T: Yes, good. (F)

[Extract Two]

T: What was Linda doing at seven yesterday? (I)

S: She was helping her mother. (R)

T: Wo*nderful.* (F)

The IR0 pattern, utilized by all six teachers, was employed when a question or task was deemed easy and comprehensible. This pattern involved an initiation by the teacher and concluded with the student's response without the provision of any feedback. Fan and Ma [45] suggest that IR0 can be considered a zero feedback discourse pattern, where the correct response from students is acknowledged. The present study finds that the IR0 pattern was commonly used for comprehensive checks, confirmation checks and even clarifications. However, the IR0 pattern may hinder learners' interest and diminish their enthusiasm for language learning. Two illustrative cases regarding the IR0 pattern are provided below.

[Extract Three]

*T: Look at the picture, which one is right? (+) (+)* (I)

*L: Picture B.* (R)

*T: (0)* (0)

[Extract Four]

*T: All right, do you understand?* (I)

*LL: Yes*. (R)

*T: (0)* (0)

**Fig. 6:**
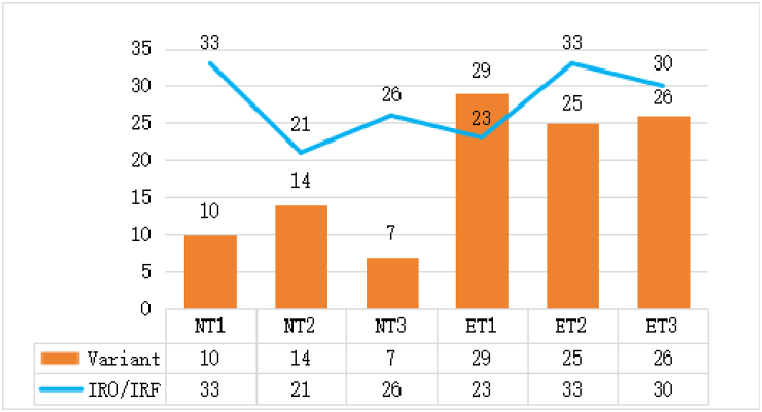
The numbers of traditional and variant discourse patterns.

To explore further, the total numbers relating to IRF/IR0 and variant patterns are presented in Fig. 6 below.

The fluctuation of IR0/IRF numbers between novice and expert teachers is vividly depicted in Fig. 6 with the curve line, showing a smooth transition between 21 and 33. However, the bar chart in Fig. 6 clearly illustrates the variation in patterns among the teachers. ET1 exhibited a peak at 29 and NT3 a low point at 7, indicating a fourfold increase in variant discourse patterns among expert teachers compared to novice teachers. The Chi-square test results shown in Table 4 support these findings. The most common variant shown in Fig. 4, [I^n^R^n^]F in blue, was prevalent among both novice (26) and expert teachers (64). Fig. 5 demonstrates that [I^n^R^n^]F accounted for 32.5 % of the total patterns, suggesting its popularity. The pattern involves the teacher initiating an exchange, prompting a related question to the student and then the teacher providing feedback. Despite variations in the number of moves, the [I^n^R^n^]F structure remained consistent in this study. Quintessential examples are as follows:

**Table 4:** Chi-square tests of discourse structures of novice versus expert teachers.

	Value	df	Asymp. Sig. (2-sided)	Exact Sig. (2-sided)	Exact Sig. (1-sided)
Pearson Chi-Square	11.375a	1	.001		
Continuity Correction	10.547	1	.001		
Likelihood Ratio	11.618	1	.001		
Fisher's Exact Test				.001	.001
Linear-by-Linear Association	11.334	1	.001		
N of Valid Cases	277				

a. 0 cells (.0 %) have expected count less than 5. The minimum expected count is 44.48.

b. Computed only for a 2x2 table.

[Extract Five]

*T: What were you doing at the time of rainstorm?* (I1)

*L: I was helping my mother.* (R1)

*T: Where? (+)* (I2)

*L: In the kitchen at home.* (R2)

*T: Wow, Great, I think you are a good boy!* (F)

[Extract Six]

*T: Okay, class. When we retell a story, try to use some linking words, understand?* (I1)

*LL: Yes.* (R1)

*T: Can you give me some linking words? (+)* (I2)

*L: So.* (R2)

*T: Anything else? (+)* (I3)

*L: Because.* (R3)

*T: Continue! Anyone? (+) (+)* (I4)

*L: And, But.* (R4)

*T: You are so smart! Besides that, you can also use firstly, secondly etc.* (F)

[Extract Seven]

*T: What did you do on weekends?* (I1)

*L: I played badminton.* (R1)

*T: When did you do that? (+) (+)* (I2)

*L: At 7 p.m. yesterday.* (R2)

*T: Okay, who did you play with?* (I3)

L: My friend, ***. (R3)

*T: So, what were you doing at 7 p.m. yesterday? (+) (+)* (I4)

L: I was playing badminton with *** at 7 p.m. yesterday. (R4)

*T: Wonderful, such a clever girl.* (F)

The IRFR and I[R^n^F^n^] patterns also play a crucial role in classroom discourse within the framework of interaction theory. The exchange of variant patterns is the process of interaction, but the teacher may adopt the IRFR pattern less frequently than other variants as it prioritizes accuracy and the introduction of new knowledge to learners. The final stage in this model involves learners repeating and imitating the information provided.

[Extract Eight]

*T: And here is a new word, can you pronounce it?* (I)

*LL: ((unintelligible))* (R)

*T: Em follow me, please! Strange.* (F)

*LL: Strange, strange.* (R)

In the I[R^n^F^n^] pattern, learners are expected to be highly engaged, as everyone can participate in the interaction and receive feedback from the teacher. This model also provides numerous opportunities for the learners to communicate and practice the target language. Two extracts serve as examples of this approach.

[Extract Nine]

*T: What were you doing at 9 p.m. last Sunday?* (I)

*L1: I was playing computer games.* (R1)

*T: So exciting!* (F1)

*L2: I was (+) (+) was hanging out with my parents.* (R2)

*T: Great, you were hanging out with your parents.* (F2)

*L3: I was doing my homework (+) at home.* (R3)

*T: Good boy!* (F3)

[Extract Ten]

*T: Who wants to retell Linda's schedule? (+) (+) (+)* (I)

*L1: She was helping her mother at seven last night.* (R1)

*T: Great!* (F1)

*L2: Linda was take a shower at eight o’ clock pm. yesterday.* (R2)

*T: Okay, Linda was (+) (+) taking a shower. Right?* (F2)

*L3: She was doing her homework when Mary called her.* (R3)

*T: Pretty good!* (F3)

Cross-tabulation and Chi-square tests were performed to analyse the traditional and variant discourse patterns among the novice and expert teachers. Table 3 displays the cross-tabulation results for IRF/IR0 and their variants, highlighting the similarities and differences in count and expected count values between the two groups. These findings were further examined through Chi-square tests, as shown in Table 4, to determine their significance.

Table 4 reveals similarities between the novice and expert teachers in the use of the traditional IRF/IR0 discourse patterns, with 80 and 86 instances, respectively. However, there was a notable contrast between the two groups in terms of variant discourse structures. Expert teachers employed variants three times more frequently (80 instances) than novice teachers did (31 instances). Specifically, the Chi-square test results in Table 4 offer a clear data analysis for the study. Importantly, these Chi-square tests were conducted for a 2 × 2 table, requiring all expected counts in the previous Table 3 to be above 5. As Table 3 shows, all expected counts exceeded 5, with the lowest expected count being 44.5, ensuring the statistical validity of the Chi-square tests. The Chi-square test results indicate a significant difference between the novice and expert teachers in terms of discourse structures (χ2 = 11.375, df = 1, Sig. (2-sided) = .001) with a p-value <.05. Furthermore, the cross-tabulation demonstrated that novice teachers tended to employ IRF/IR0 patterns more than expert teachers did, as the count exceeded the expected count (66.5) by 80 instances. At the same time, expert teachers favoured variant discourses, as the count data surpassed the expected count. This aligns with the previous analyses and interviews, highlighting that expert teachers were more inclined than novice teachers to adopt variant discourse patterns. It is also evident that novice teachers often underestimated the significance of discourse patterns, with many proposed turn-taking mechanisms lacking supporting evidence from the interviews.

In addition to Li and Fan's [33] classification, another variant pattern was identified in this research, referred to as IR^n^F (see Extract Eleven). This creative approach was observed 11 times across the classes of the three expert teachers—five times more frequently than the novice teachers. This pattern typically occurred during group work, where the teacher initiated by asking the learners to share ideas or engage in role play within their groups. Subsequently, certain group members were expected to respond without any intervention by the teacher, and then the teacher would evaluate their performance. Working in groups is undeniably beneficial for learners as it promotes class engagement and independent thinking. Moreover, group work fosters teamwork and competitive consciousness among learners. Collaborating closely with classmates also helps learners become more attuned to their own strengths and weaknesses through a critical evaluation of their peers' work. Such activities not only enhance language proficiency but also align with the core competencies outlined in the national curriculum. Additionally, explaining and defending ideas to peers in group settings not only clarifies and refines learners' concepts but also encourages critical thinking.

[Extract Eleven]

*T: It is show time! Which group wants to show? (+) (+) Wow, excellent! You, please!* (I)

*L: Were you eating dinner at 6 p.m. yesterday?* (R)

*L: Yes, I was. Were you doing your homework at 6 p.m. yesterday?* (R)

*L: No, I was not. I was watching TV at that time. Were you playing soccer at 6 p.m. yesterday?* (R)

*L: No. I was not. I was play (+) (+) playing computer games at that time.* (R)

*T: Wonderful! Congratulation, group five!* (F)

The data and comparison between novice and expert teachers yielded the following findings. First, the IRF/IR0 patterns, the basic structure, prevailed and were commonly used in EFL classes, aligning with Zhao [46], as they serve as basic turn-taking units facilitating easy initiation and conclusion for the teacher while also being less time-consuming compared to other variants. This teacher-oriented approach continues to dominate in EFL classrooms, explaining the prevalence of IRF/IR0 patterns. Second, distinctions between novice and expert teachers emerged when examining variant structures. Expert teachers tended to use the IRFR structure three times more frequently than novice teachers did, with IRFR focusing on student responses rather than teacher feedback and often concluding with repetition or imitation, as highlighted by Liu [40], who emphasizes imitation as a valuable SLA strategy. Despite its effectiveness, the IRFR pattern is less common compared to other variants in the research corpus. The variant patterns [I^n^R^n^]F and I[R^n^F^n^] display significant differences between novice and expert teachers, with students in the expert teachers’ classes displaying more positive results due to the complex and varied patterns adopted. The use of interactive and communicative patterns, such as [I^n^R^n^]F and I[R^n^F^n^], can provide more opportunities for students, fostering a sense of achievement. It is important to recognize that learners must develop skills beyond just intellectual ones, and one effective way to achieve this is through active engagement and close collaboration with teachers. These patterns involve various moves that encourage interaction and communication among learners, leading to a positive impact on their learning outcomes. They also help learners develop judgement and critical thinking skills through exchanges. In contrast, basic discourse patterns may stifle creativity and lead to frustration. Specifically, the [I^n^R^n^]F and I[R^n^F^n^] patterns offer opportunities for students to actively participate in class, develop independent learning and acquire problem-solving skills. These competencies are central to the new national curriculum, which aims to produce students who are not only proficient in English but also capable of using the language in real-world contexts. However, fixed patterns (IRF/IR0) may limit learner engagement and hinder autonomous learning abilities. Therefore, teachers are expected to adopt a production-oriented approach, emphasizing the importance of offering diverse perspectives and stimulating critical thinking to empower students. The findings from some interviews support this conclusion.

(1) *In class, I tend to speak more than the students in order to facilitate the learning process. I typically employ a question-and-answer (IRF pattern) method that not only saves time but also helps me gauge student understanding quickly. However, in activities such as role-playing or group work, I refrain from using a question-and-answer approach. By promoting more interactive discourse (variant patterns), students are provided with more opportunities to enhance their foreign language learning.* (NT1, a 25-year-old female)
(2) *I am accustomed to providing students with ample opportunities for self-expression, encourage peer communication, and offer support when they face challenges. I believe that cultivating interest and enthusiasm is crucial for middle school students learning a foreign language. To sustain their motivation, it is essential to prioritize student growth and ensure they engage in sufficient language output.* (ET2, a 43-year-old female)

The interviews with novice and expert teachers provided qualitative insights into the factors contributing to these disparities relating to the third research question. Novice teachers reported a preference for the IRF pattern due to its simplicity and efficiency in maintaining order in the classroom. However, NT1 acknowledged its limitations in promoting learners' interaction. Expert teachers emphasized the importance of providing learners with opportunities for self-expression and peer communication. ET2 highlighted the effectiveness of interactive discourse patterns in maintaining the learners' interest and facilitating language acquisition. Expert teachers’ use of variant structures was associated with higher student engagement and a more dynamic classroom environment. As previously discussed, the notable differences between the discourse patterns of novice and expert teachers can be attributed to several factors. First, teaching ideologies play a critical role. Expert teachers are more likely to adopt learner-centred approaches that prioritize interaction and engagement, in contrast to the more instructor-centred methods often employed by novices. Second, proficiency in the target language significantly influences discourse practices. Expert teachers, with their higher language proficiency, are better equipped to manage and diversify classroom interactions, thus providing richer linguistic input. Third, classroom management skills are pivotal. Experienced teachers possess superior classroom management abilities, allowing them to seamlessly integrate various discourse patterns without risking disorder in the classroom.

## 5 Conclusion

This study delved into the intricate dynamics of teacher discourse in junior high school EFL classrooms in China, offering a comparative analysis of novice and expert teachers. The findings reveal that while IRF and IR0 are the predominant patterns across both groups. Novice teachers tended to rely heavily on the traditional IRF and IR0 patterns, which, though effective in maintaining order in the classroom, may limit opportunities for extended discourse and student engagement. On the contrary, expert teachers exhibited a greater propensity for employing variant discourse patterns, such as IRFR, [I^n^R^n^]F and I[R^n^F^n^]. These patterns foster a more interactive and engaging learning environment, thereby enhancing student participation and language acquisition. At the same time, the expert teachers' ability to adapt their discourse strategies and employ more complex patterns, particularly the novel IR^n^F pattern identified in this research, significantly contributed to production-oriented group work settings. Such adaptability not only facilitates deeper interaction but also promotes critical thinking and independent learning among students. Specifically, the findings of this study highlight the significant impact of teacher discourse patterns on students' foreign language learning. The increased opportunities for interaction was the basic contribution of the teacher discourse patterns. Expert teachers tended to use varied discourse patterns, such as IRFR, [I^n^R^n^]F and I[R^n^F^n^], and these patterns increased the opportunities for students to respond and receive feedback, thus facilitating deeper interaction and understanding. Fostering the development of thinking skills was the second contribution of the teacher discourse patterns. Varied discourse patterns, especially the variant patterns, encourage students to engage in critical thinking and independent thought. For instance, the IRFR pattern reinforces students' grasp of new knowledge through repetition and imitation, while the I[R^n^F^n^] pattern promotes teamwork and collective intelligence through multiple student interactions and feedback. The third contribution of the teacher discourse patterns was the improvement in language fluency and accuracy. The study shows that expert teachers' use of complex discourse patterns provides students with more opportunities for language practice, thereby improving both their fluency and accuracy. For example, the [I^n^R^n^]F pattern involves continuous questioning and feedback, helping students use the target language more accurately and reinforcing their learning through repetition. The finding of increased interest and motivation is the last contribution of this study. Expert teachers' use of highly interactive and challenging discourse patterns successfully stimulates learners’ interest and motivation to learn. By engaging in these interactions, learners experience a sense of achievement and self-worth, leading to more active and motivated participation in language learning.

In light of these findings, this study has valuable implications for teacher professional development. It advocates for the integration of varied and interactive discourse structures in EFL teaching, emphasizing the need for continuous training and support for novice teachers to develop their discourse strategies. By bridging the gap between theoretical frameworks and practical application, this research contributes to the development of a broader understanding of effective teaching practices in junior high school EFL contexts. This study provides several important insights for teachers to master diverse teaching strategies and establish reflective teaching practices. This is particularly crucial for novice teachers, who should receive systematic training to learn and master various classroom discourse patterns. Additionally, they must engage in regular self-reflection and receive peer feedback to improve their discourse patterns.

Although this study was conducted meticulously from start to finish, it is important to acknowledge its limitations due to constraints, such as time and other conditions. Firstly, it must be noted that this research focused exclusively on female teachers’ interactions. Gender is a crucial factor in research as different genders may approach and execute tasks differently, leading to varying outcomes. Therefore, as the study excluded male teachers, the comparative results of this study may not be generalizable to all novice and expert teachers. Secondly, the study included only six teachers from junior high schools, resulting in a relatively small sample size and limited scope. This small sample may not have fully captured the characteristics of novice and expert teachers. Additionally, despite the multiple rounds of transcription that took place, some words may have been unintelligible due to recording equipment limitations, potentially impacting the analysis.

Despite these limitations, this study provides valuable insights into the challenges faced by novice teachers regarding teacher discourse. While this was a comprehensive study, there remain unexplored areas due to the aforementioned limitations. In light of advancements in technology, one emerging suggestion in the field of education is to implement flipped classrooms, which prioritize a learner-centred approach. Therefore, future research should focus on key questions, for instance, about the characteristics of teacher discourse within flipped classrooms and the differences in teachers’ discourse quantity in traditional versus flipped classroom settings. These research avenues warrant further investigation in future studies.

## Ethics statement

All methods were carried out in accordance with the guidelines and regulations of the 1964 Helsinki declaration and its later amendments. Since this study does not involve intervention and it is low risk, ethical review and approval were waived according to the institutional review boards at School of Foreign Languages in Yanbian University.

And the written informed consent was obtained from all participants (including the parental consent is also required for research including under-18s) in terms of observation for this study. All participants were informed about the aim of the study and their right to withdraw from the study at any time. All methods were carried out in accordance with relevant guidelines and regulations.

## Funding

This research did not receive any specific grant from funding agencies in the public, commercial, or not-for-profit sectors.

## Data availability statement

The data of this study is not deposited into a publicly available repository, because the data that has been used is confidential. For further access please contact the corresponding author.

## CRediT authorship contribution statement

**Zhiyue Tong:** Writing – review & editing, Writing – original draft, Resources, Investigation, Formal analysis. **Fengcun An:** Methodology, Investigation, Formal analysis. **Yanji Cui:** Writing – review & editing.

## Declaration of competing interest

The authors declare the following financial interests/personal relationships which may be considered as potential competing interests: Fengcun An reports was provided by Yanbian University. Fengcun An reports a relationship with Yanbian University that includes: employment. If there are other authors, they declare that they have no known competing financial interests or personal relationships that could have appeared to influence the work reported in this paper.
